# A Cirurgia é o Meio e não o Desfecho: Seguimento Tardio da Cirurgia de Revascularização Miocárdica

**DOI:** 10.36660/abc.20260351

**Published:** 2026-05-26

**Authors:** João Carlos Ferreira Leal, Rafael Alessandro Ferreira Gomes, Walter J. Gomes

**Affiliations:** 1 Faculdade Estadual de Medicina do Estado de São Paulo São José do Rio Preto SP Brasil Faculdade Estadual de Medicina do Estado de São Paulo, São José do Rio Preto, SP – Brasil; 2 Universidade de Pernambuco Recife PE Brasil Universidade de Pernambuco, Recife, PE – Brasil; 3 Escola Paulista de Medicina Universidade Federal de São Paulo São Paulo SP Brasil Escola Paulista de Medicina – Universidade Federal de São Paulo, São Paulo, SP – Brasil

**Keywords:** Doença da Artéria Coronariana, Revascularização Miocárdica, Revascularização Miocárdica, Prognóstico

A cirurgia de revascularização do miocárdio (CRM) permanece em posição de destaque no tratamento da doença arterial coronariana (DAC), sobretudo nos subgrupos de pacientes de maior risco, como doença de múltiplos vasos, disfunção ventricular esquerda, diabetes mellitus e carga isquêmica elevada, comprovada por décadas de evidências científicas e seus resultados consistentes em longo prazo.

A DAC tem evolução progressiva, sistêmica e crônica, o que torna o seguimento em longo prazo, essencial para a interpretação dos resultados nas estratégias terapêuticas. Desta forma, os resultados após a CRM não podem se restringir apenas ao sucesso técnico do procedimento nem aos desfechos intra-hospitalares, e sim à perspectiva mais ampla e integrativa em longo-prazo.

O estudo “Mortalidade em Cinco Anos em Sobreviventes de Cirurgia de Revascularização do Miocárdio: Um Estudo Retrospectivo de um Centro Terciário”^1^ traz contribuições relevantes ao explorar o risco residual e os determinantes prognósticos tardios após CRM.

Os autores analisaram 6.933 pacientes submetidos a CRM com seguimento médio de quatro anos e 31.610 pacientes-ano de observação, e mostraram que a mortalidade por todas as causas em até cinco anos foi de 12,9%, enquanto a mortalidade cardiovascular alcançou 5,7%. Entre os óbitos cardiovasculares, a doença isquêmica do miocárdio foi predominante com 57,3%, seguida pela doença cerebrovascular.^1^ Estes resultados demonstram números relevantes, sendo que a mortalidade em longo prazo após a CRM geralmente varia de 10% a 20% em 5 anos, dependendo das características do paciente e da complexidade da doença.

É importante ressaltar que o estudo FREEDOM demonstrou a superioridade da CRM em pacientes diabéticos com doença de múltiplos vasos, demonstrando uma redução significativa em eventos cardiovasculares adversos maiores e mortalidade.^2^ Da mesma forma, o acompanhamento estendido do estudo SYNTAX destacou a importância da complexidade anatômica, com a CRM mantendo uma vantagem de sobrevida em pacientes com escores SYNTAX mais elevados.^3^ Estes estudos reforçam que a CRM é o tratamento que muda prognóstico dos pacientes com DAC e que os resultados em longo prazo são determinados pela interação entre o perfil de risco do paciente, as características anatômicas e durabilidade da revascularização. O benefício cirúrgico precisa ser associado por prevenção secundária intensiva, controle de fatores de risco e adesão medicamentosa otimizada.

Os fatores de risco que associaram à evolução desfavorável nos pacientes analisados foram a faixa etária avançada, diabetes mellitus, função renal e fração de ejeção reduzidas, aumento das cavidades atrial e ventricular esquerda, internação de urgência, maior permanência hospitalar e cirurgia sem circulação extracorpórea (CEC). A cirurgia sem CEC neste estudo foi associada como fator desfaforável, entretanto em outros estudos os resultados foram satisfatórios. Os parâmetros ecocardiográficos, átrio esquerdo aumentado e a disfunção ventricular, reforçam a presença do remodelamento miocárdico, sobrecarga hemodinâmica e maior vulnerabilidade a insuficiência cardíaca, fibrilação atrial e novos eventos cardíacos. Neste contexto, a função renal tem associação não linear entre taxa de filtração glomerular estimada e mortalidade, com incremento mais acentuado do risco abaixo de 60 mL/min/1,73 m^2^, reforça a gravidade da doença renal crônica na trajetória do paciente após a CRM.^4,5^

A associação entre CRM exclusivamente com enxertos arteriais e redução da mortalidade cardiovascular, destaca a importância do enxerto arterial como fator prognóstico em longo prazo. Embora ensaios randomizados como o ART não tenham demonstrado uma clara vantagem de sobrevida para o enxerto bilateral da artéria torácica interna,^6^ evidências acumuladas de estudos observacionais e meta-análises sugerem que a CRM somente com enxertos arteriais pode conferir benefícios a longo prazo em pacientes selecionados.^7^ Esta informação mostra que a escolha do enxerto é determinante no resultado.

Em termos práticos, este estudo avalia aspectos relevantes que interferem no prognóstico da CRM e mostra a necessidade de assistência dos pacientes em longo prazo. O retorno destes pacientes ao ambulatório após hospitalização complicada deve ser visto não como sobreviventes estáveis, mas como indivíduos em fase de risco ainda ativo. A CRM com sucesso vai além do próprio procedimento, ela é o meio e não o desfecho. Depende da integração da correção anatômica, do manejo clínico e do acompanhamento multidisciplinar contínuo.
