# Supplementary material for: A Cirurgia é o Meio e não o Desfecho: Seguimento Tardio da Cirurgia de Revascularização Miocárdica
Source: Arq Bras Cardiol. 2026 May 26;123(4):e20260351. [Article in Portuguese] doi: 10.36660/abc.20260351 (PMC13398846; doi:10.36660/abc.20260351)
Supplement: Translation

# Translation: Surgery is the Means, Not the End: Late Follow-Up of Coronary Artery Bypass Graft Surgery

**DOI:** 10.36660/abc.20260351i

**Authors:** João Carlos Ferreira Leal, Rafael Alessandro Ferreira Gomes, Walter J. Gomes

**Affiliations:** 1 Brasil Faculdade Estadual de Medicina do Estado de São Paulo, São José do Rio Preto, SP – Brasil; 2 Brasil Universidade de Pernambuco, Recife, PE – Brasil; 3 Brasil Escola Paulista de Medicina – Universidade Federal de São Paulo, São Paulo, SP – Brasil

**Keywords:** Coronary Artery Disease, Myocardial Revascularization, Myocardial Revascularization, Prognosis

Coronary artery bypass grafting (CABG) remains a leading treatment for coronary artery disease (CAD), particularly in high-risk patient subgroups such as those with multivessel disease, left ventricular dysfunction, diabetes mellitus, and high ischemic burden, as evidenced by decades of scientific evidence and its consistent long-term results.

Coronary artery disease (CAD) has a progressive, systemic, and chronic evolution, which makes long-term follow-up essential for interpreting the results in therapeutic strategies. Therefore, outcomes after coronary artery bypass grafting (CABG) cannot be limited solely to the technical success of the procedure or to in-hospital outcomes but must encompass a broader and more integrative long-term perspective.

The study “Five-Year Mortality among Survivors of Coronary Artery Bypass Graft Surgery: A Retrospective Study from a Tertiary Center”^1^ makes relevant contributions by exploring residual risk and late prognostic determinants after CABG.

The authors analyzed 6,933 patients who underwent CABG with a mean follow-up of four years and 31,610 patient-years of observation and showed that all-cause mortality within five years was 12.9%, while cardiovascular mortality reached 5.7%. Among cardiovascular deaths, ischemic heart disease was predominant at 57.3%, followed by cerebrovascular disease.^1^ These results demonstrate relevant figures, with long-term mortality after CABG generally ranging from 10% to 20% over 5 years, depending on patient characteristics and disease complexity.

It is important to highlight that the FREEDOM study demonstrated the superiority of CABG in diabetic patients with multivessel disease, showing a significant reduction in major adverse cardiovascular events and mortality.^2^ Similarly, the extended follow-up of the SYNTAX study highlighted the importance of anatomical complexity, with CABG maintaining a survival advantage in patients with higher SYNTAX scores.^3^ These studies reinforce that CABG is the prognosis-changing treatment for patients with CAD and that long-term outcomes are determined by the interaction between the patient’s risk profile, anatomical characteristics, and durability of revascularization. The surgical benefit needs to be combined with intensive secondary prevention, control of risk factors, and optimized medication adherence.

The risk factors associated with unfavorable outcomes in the patients analyzed were advanced age, diabetes mellitus, reduced renal function and ejection fraction, enlarged left atrial and ventricular chambers, emergency hospitalization, longer hospital stay, and surgery without cardiopulmonary bypass (CPB). In this study, surgery without CPB was associated as an unfavorable factor; however, other studies have shown satisfactory results. Echocardiographic parameters, including an enlarged left atrium and ventricular dysfunction, reinforce the presence of myocardial remodeling. Hemodynamic overload and increased vulnerability to heart failure, atrial fibrillation, and new cardiac events. In this context, renal function has a non-linear association between estimated glomerular filtration rate and mortality, with a more pronounced increase in risk below 60 mL/min/1.73 m^2^, reinforcing the severity of chronic kidney disease in the patient’s trajectory after CABG.^4,5^

The association between CABG exclusively with arterial grafts and reduced cardiovascular mortality highlights the importance of arterial grafting as a long-term prognostic factor. Although randomized trials such as ART have not demonstrated a clear survival advantage for bilateral internal thoracic artery grafting,^6^ accumulated evidence from observational studies and meta-analyses suggests that CABG with arterial grafts alone may confer long-term benefits in selected patients.^7^ This information shows that graft choice is crucial to the outcome.

In practical terms, this study evaluates relevant aspects that interfere with the prognosis of CABG and shows the need for long-term patient care. The return of these patients to the outpatient clinic after complicated hospitalization should be viewed not as stable survivors, but as individuals still at active risk. CABG with success goes beyond the procedure itself; it is the means, not the end. It depends on the integration of anatomical correction, clinical management, and continuous multidisciplinary follow-up.
